# Nutritional status and survival of 8247 cancer patients with or without diabetes mellitus—results from a prospective cohort study

**DOI:** 10.1002/cam4.3397

**Published:** 2020-08-19

**Authors:** Minghua Cong, Wenjie Zhu, Chang Wang, Zhenming Fu, Chunhua Song, Zhong Dai, Keqing Yao, Zengqing Guo, Yuan Lin, Yingying Shi, Wen Hu, Yi Ba, Suyi Li, Zengning Li, Kunhua Wang, Jing Wu, Ying He, Jiajun Yang, Conghua Xie, Xinxia Song, Gongyan Chen, Wenjun Ma, Suxia Luo, Zihua Chen, Hu Ma, Chunling Zhou, Wei Wang, Qi Luo, Yongmei Shi, Yumei Qi, Haiping Jiang, Wenxian Guan, Junqiang Chen, Jiaxin Chen, Yu Fang, Lan Zhou, Yongdong Feng, Rongshao Tan, Tao Li, Junwen Ou, Qingchuan Zhao, Jianxiong Wu, Li Deng, Xin Lin, Liuqing Yang, Hongxia Xu, Wei Li, Lei Yu, Hanping Shi, Investigation on Nutrition Status, Clinical Outcome of Common Cancers (INSCOC) Group

**Affiliations:** ^1^ Department of Comprehensive Oncology National Cancer Center/National Clinical Research Center for Cancer/Cancer Hospital Chinese Academy of Medical Sciences and Peking Union Medical College Beijing China; ^2^ Cancer Center of the First Hospital of Jilin University Changchun China; ^3^ Cancer Center Renmin Hospital of Wuhan University Wuhan China; ^4^ Department of Epidemiology College of Public Health Zhengzhou University Zhengzhou China; ^5^ Department of Comprehensive Oncology Huanxing Cancer Hospital Beijing China; ^6^ Department of Medical Oncology Fujian Cancer Hospital Fujian Medical University Cancer Hospital Fuzhou China; ^7^ Department of Gastrointestinal Surgery Affiliated Tumor Hospital of Guangxi Medical University Nanning China; ^8^ Department of Surgery The First Affiliated Hospital of SunYat‐sen University Guangzhou China; ^9^ Department of Clinical Nutrition West China Hospital of Sichuan University Chengdu China; ^10^ Department of Gastrointestinal Oncology Tianjin Key Laboratory of Cancer Prevention and Therapy Tianjin Medical University Cancer Institute and Hospital National Clinical Research Center for Cancer Tianjin China; ^11^ Department of Nutrition and Metabolism of Oncology Affiliated Provincial Hospital of Anhui Medical University Hefei China; ^12^ Department of Clinical Nutrition The First Hospital of Hebei Medical University Shijiazhuang China; ^13^ Department of Gastrointestinal Surgery Institute of Gastroenterology The First Affiliated Hospital of Kunming Medical University Kunming China; ^14^ Department of Clinical Nutrition The First People's Hospital of Kashi Xinjiang China; ^15^ Department of Clinical Nutrition Chongqing General Hospital Chongqing China; ^16^ Department of Colorectal and Anal Surgery Huizhou Municipal Central Hospital Huizhou China; ^17^ Department of Radiation and Medical Oncology Zhongnan Hospital of Wuhan University Wuhan China; ^18^ Department of Oncology Xingtai People’s Hospital Hebei Medical University Xingtai China; ^19^ The First Department of the Tumor Hospital of Harbin Medical University Harbin China; ^20^ Department of Nutrition Guangdong General Hospital Guangdong Academy of Medical Sciences Guangzhou China; ^21^ Department of Oncology Affiliated Cancer Hospital of Zhengzhou University and Henan Cancer Hospital Zhengzhou China; ^22^ Department of General Surgery Xiangya Hospital Central South University Changsha China; ^23^ Department of Oncology Affiliated Hospital of Zunyi Medical University Zunyi China; ^24^ The Fourth Affiliated Hospital Harbin Medical University Harbin China; ^25^ Cancer Center The First People's Hospital of Foshan Foshan China; ^26^ Department of Gastrointestinal Tumor Surgery The First Affiliated Hospital Xiamen University Xiamen China; ^27^ Department of Nutrition Ruijin Hospital Shanghai Jiao Tong University School of Medicine Shanghai China; ^28^ Department of Nutrition Tianjin Third Central Hospital Tianjin China; ^29^ Department of Surgery The First Affiliated Hospital of Jinan University Guangzhou China; ^30^ Department of General Surgery Nanjing Drum Tower Hospital The Affiliated Hospital of Nanjing University Medical School Nanjing China; ^31^ Department of Gastrointestinal Surgery First Affiliated Hospital of Guangxi Medical University Nanning China; ^32^ Department of Radiation and Medical Oncology People's Hospital of Guangxi Zhuang Autonomous Region Nanning China; ^33^ Department of Clinical Nutrition Peking University Cancer Hospital and Institute Beijing China; ^34^ Department of Nutrition Third Affiliated Hospital of Kunming Medical College Tumor Hospital of Yunnan Province Kunming China; ^35^ Department of Surgery Tongji Hospital Tongji Medical College Huazhong University of Science and Technology Wuhan China; ^36^ Department of Nutrition Guangzhou Red Cross Hospital Guangzhou China; ^37^ Department of Radiotherapy Sichuan Cancer Hospital & Institute Sichuan Cancer Center School of Medicine University of Electronic Science and Technology of China Chengdu China; ^38^ Department of Clinical Nutrition Clifford Hospital Guangzhou University of Chinese Medicine Guangzhou China; ^39^ Department of Digestive Diseases Xijing Hospital Fourth Military Medical University Xi'an China; ^40^ Department of Hepatobiliary Surgery National Cancer Center/Cancer Hospital Chinese Academy of Medical Sciences and Peking Union Medical College Beijing China; ^41^ Department of Nutrition Daping Hospital & Research Institute of Surgery Third Military Medical University Chongqing China; ^42^ Department of Gastrointestinal Surgery/Clinical Nutrition Beijing Shijitan Hospital Capital Medical University The 9th Clinical College Beijing China

**Keywords:** cancer, diabetes mellitus, handgrip, malnutrition, nutritional status

## Abstract

**Background:**

The number of cancer patients with diabetes mellitus (DM) is steadily rising. Little is known about the nutritional status of this population. This study characterized the nutritional status and survival of cancer patients with diabetes compared with those without diabetes.

**Methods:**

A total of 8247 cancer patients were prospectively enrolled from 72 hospitals in China and followed until August 2019. A global estimation of the nutritional status was performed for each participant using standardized tools. The outcomes were cancer‐specific survival (CSS) and overall survival (OS).

**Results:**

The incidence of diabetes was 7.6% in the whole population. In comparison with the non‐DM group, the DM group had greater body weight, but a similar fat‐free mass, a lower handgrip strength and a decreased Karnofsky performance score. A higher proportion of patients with diabetes were overweight/obese as indicated by BMI. The percentage of patients who were at risk of malnutrition (evaluated by PG‐SGA) was higher in the DM group (score ≥ 4, 56.7% vs 52.9%). Patients with DM showed a worse CSS (4‐year CSS, 62% vs 73%) and OS (4‐year OS 39% vs 52%). Diabetes was associated with an increased risk of both cancer‐specific (hazard ratio (HR) = 1.282, 95% confidence interval (CI) 1.070‐1.536) and overall (HR = 1.206, 95% CI 1.040‐1.399) mortality.

**Conclusions:**

Cancer patients with diabetes had a larger body mass but lower muscle strength, poorer performance status and higher incidence of malnourishment. Diabetes was associated with compromised survival. Tailored nutritional intervention is necessary for this subpopulation of patients.

## Graphical abstract

In this large prospective cohort study, cancer patients with diabetes were found to have bigger body mass yet lower muscle strength, poorer performance status and higher incidence of malnourishment. Complication with diabetes independently predicted compromised survival among cancer patients.

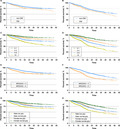

## 1 INTRODUCTION

Cancer poses a major threat to global health and has become a rapidly growing burden to the medical care system in China. There were an estimated 4.3 million new cancer cases and 2.9 million cancer deaths in 2018 in China.^1^

Nutritional disorders are prevalent in patients with malignant disease. It has been reported that 8%‐87% of patients with malignancy develop undernutrition, or malnutrition by deficit, at some point during the course of the disease.^2^, ^3^, ^4^ Undernutrition is of clinical concern given its acknowledged influence over antineoplastic treatment and prognosis. The real picture however is far more complicated. An increasing body of evidence suggests that excess body weight/obesity is a risk factor for cancer,^5^ and obese patients with cancer have a compromised survival compared with those of normal weight.^6^, ^7^, ^8^ Thus, malnutrition by excess is drawing increasing attention in clinical practice.

The presence of comorbid medical conditions other than cancer further complicates the diverging patterns of nutritional status described above. Diabetes mellitus (DM) is a common complication in cancer patients. It has been well established that overweight and obese patients are at higher risk of developing diabetes.^9^ Yet patients with poor glycemic control often experience weight loss and emaciation due to impaired glucose metabolism and the compensatory consumption of lipids and protein. These metabolic alterations may account for the varying nutritional status observed in patients with diabetes. Of note, the number of cancer patients complicated with diabetes has been steadily increasing in recent years. Nevertheless, little insight has been provided into the nutritional status of this population. Therefore, the current study evaluated the nutritional status of cancer patients with or without diabetes using diverse methods of nutritional assessment. We then compared the survival status of patients with diabetes to that of patients without.

## 2 METHODS

### 2.1 Participants and data collection

This was a prospective cohort study based on the Investigation on Nutritional Status and its Clinical Outcomes of Common Cancers (INSCOC), which was a multi‐center cross‐sectional observational study with linked follow‐up data. Patients were consecutively enrolled from 72 hospitals in China from July 2015 to April 2018. Adult patients were eligible if they were pathologically diagnosed with malignant disease and admitted for anti‐cancer treatment (surgery, radiotherapy, chemotherapy, etc). All participants provided written informed consent. The protocol was approved by the INSCOC Research Ethics Committee and the Institutional Review Boards of all participating institutions.

For each subject, demographic and clinical information was recorded by trained medical staff who filled in pre‐constructed forms. On admission into the hospital, interviews were performed to collect data for each patient including the age, sex, drinking/smoking status, degree of education, area of residence, history of past illness, family history of cancer, cancer‐related variables, and anti‐cancer treatment. For comorbidities, we focused on DM, which was defined as meeting one of the following criteria: (a) a previous diagnosis of diabetes, (b) a fasting plasma glucose level of ≥7.0 mmol/L, (c) 2‐hr plasma glucose level of ≥11.1 mmol/L by the oral glucose tolerance test, (d) a glycated hemoglobin (HbA1c) level of ≥6.5%, (e) a random glucose level of ≥11.1 mmol/L with typical symptoms related to high blood glucose. All participants were grouped at baseline by whether they were complicated with DM.

To provide a global estimate of the nutritional status, a series of parameters were investigated including height, weight, body mass index (BMI), handgrip, fat‐free mass, and blood test indices such as hemoglobin, pre‐albumin, albumin, and C‐reactive protein (CRP) levels. Measurements were collected from fasting patients in the morning, who were shoeless and wore lightweight clothing. A floor scale with an incorporated stadiometer was used to determine the patient's weight and height, which were then utilized to calculate BMI (weight [kg]/height^2^ [m^2^]). A BMI of <18.5 indicated undernutrition, 18.5‐24.9 normal status, 25‐29.9 overweight and ≥30.0 obesity. Handgrip was measured on the non‐dominant hand using an electronic hand dynamometer (EH101; CAMRY). According to the Asian Working Group for Sarcopenia (AWGS) criterion, low muscle strength was defined as <18 kg for women and <26 kg for men.^10^ Data for fat‐free mass were obtained using an Inbody770 body composition analyzer (Korea). We adopted common tools for the assessment of nutritional risk, including nutrition risk screening 2002 (NRS2002) and patient‐generated subjective global assessment (PG‐SGA). For each tool, a total score was obtained which categorized the patients into different types of nutritional status. An NRS2002 score ≥3 indicated nutritional risk while a score <3 suggested no risk. PG‐SGA score classified each participant into one of the following categories: (a) 0‐1, well‐nourished; (b) 2‐3, suspected malnutrition; (c) 4‐8, moderately malnourished; (d) ≥9, severely malnourished. We organized intensive training on standardized data collection and measurements and performed regular quality assessment prior to and during the study in order to minimize the information bias.

### 2.2 Sample size and statistical analysis

Endpoints were cancer‐specific survival (CSS) and overall survival (OS). CSS was determined from the date of study enrollment to the date of cancer‐specific death or last follow‐up. OS was calculated from the date of study enrollment to the date of all‐cause death or last follow‐up. Relative differences >30% in survival proportions between groups were considered to be clinically relevant. According to data from previous studies, the five‐year survival rates for cancer patients with/without DM were 31% and 45%, respectively.^11^ Using a two‐sided log‐rank test, a sample size of 491 was found to be sufficient to detect a relative difference of 45% in 5‐year survival rates between DM and non‐DM groups at an α‐level of 5% with a power of 95%.

The Chi‐squared test was performed to compare the distribution of patient characteristics and nutritional status between the DM and non‐DM groups. Values for all nutritional parameters were expressed as numerical variables as appropriate, and *t*‐tests were conducted for inter‐group comparisons. The Kaplan‐Meier method was adopted to depict the survival curves for participants, and inter‐group comparisons were performed by log‐rank tests. The prognostic value of DM and other potential covariates was assessed using univariate analysis by the log‐rank test, and multivariate Cox proportional hazards regression model was constructed to control for confounders. All *P* values reported were two‐sided, with *P* < .05 considered statistically significant. All statistical analyses were performed with SPSS version 19.0 (SPSS Company).

## 3 RESULTS

### 3.1 Patient characteristics

A total of 8697 patients were prospectively assessed. Of these, 433 were determined to have benign disease by pathological examination and were excluded. Another 17 patients were excluded due to incomplete information. Finally, a total of 8247 patients with 16 types of malignant tumors were included in the current study (Figure S1). All participants provided complete series of data on related variables and were prospectively followed up from the date of enrollment until death or August 2019.

52.0% of the study population was male, with a median age of 55 (range, 18‐115) years. The distribution of different cancers is depicted in Figure S2, with colorectal (n = 1630, 19.8%), lung (n = 1571, 19.0%), and breast cancer (n = 1245, 15.1%) ranking as the most common malignancies. This finding was largely in agreement with the cancer statistics of the Chinese population, indicating the relatively good representativeness of the sampled cohort. The incidence of diabetes was 7.6% (624/8247) in the whole population. When further stratified by cancer types (Figure S3), it was found that diabetes was most prevalent in patients with cancers of the pancreas (16.2%), colon and rectum (9.9%), liver (7.8%) and lung (7.8%).

The subjects were divided into diabetes mellitus (DM) and non‐DM groups based on the presence/absence of diabetes. The baseline characteristics of both groups are summarized in Table 1. In comparison with the non‐DM group, the DM group comprised more male subjects (56.4% vs 51.6%). Cancer‐related variables such as the TNM staging and drinking/smoking status were not significantly different between the groups. Patients with diabetes tended to be worse educated, urban residents, and had a higher incidence of medical comorbidities.

**TABLE 1:** Baseline characteristics of cancer patients with and without diabetes mellitus

Characteristics	Groups		Total	*P*‐value
	Non‐DM (n/%)	DM (n/%)		
Sex				
Male	3936 (51.6)	352 (56.4)	4288 (52.0)	**.022**
Female	3687 (48.4)	272 (43.6)	3959 (48.0)	
TNM stage				
Unknown	108 (1.4)	8 (1.3)	116 (1.4)	.341
I	1034 (13.6)	86 (13.8)	1120 (13.6)	
II	1787 (23.4)	153 (24.5)	1940 (23.5)	
III	2650 (34.8)	233 (37.3)	2883 (35.0)	
IV	2044 (26.8)	144 (23.1)	2188 (26.5)	
Primary lesion				
Absent	3531 (46.3)	313 (50.2)	3844 (46.6)	.066
Present	4092 (53.7)	311 (49.8)	4403 (53.4)	
Drinking status				
Occasional/Never	6244 (81.9)	506 (81.1)	6750 (81.8)	.627
Regular	1379 (18.1)	118 (18.9)	1497 (18.2)	
Smoking status				
Occasional/Never	4634 (60.8)	380 (60.9)	5014 (60.8)	.966
Regular	2989 (39.2)	244 (39.1)	3233 (39.2)	
Area of residence				
Urban	4685 (61.5)	485 (77.7)	5170 (62.7)	**<.001**
Rural	2938 (38.5)	139 (22.3)	3077 (37.3)	
Degree of education				
(Under)graduate	6495 (85.2)	496 (79.5)	6991 (84.8)	**<.001**
Primary/middle/home schooling	1128 (14.8)	128 (20.5)	1256 (15.2)	
Family history of cancer				
None	6533 (85.7)	521 (83.5)	7054 (85.5)	.139
Yes	1090 (14.3)	103 (16.5)	1193 (14.5)	
Chronic pancreatitis				
No	7616 (99.9)	622 (99.7)	8238 (99.9)	.144
Yes	7 (0.1)	2 (0.3)	9 (0.1)	
Hypertension				
No	6532 (85.7)	359 (57.5)	6891 (83.6)	**<.001**
Yes	1091 (14.3)	265 (42.5)	1356 (16.4)	
Coronary heart disease				
No	7379 (96.8)	551 (88.3)	7930 (96.2)	**<.001**
Yes	244 (3.2)	73 (11.7)	317 (3.8)	
Stroke				
No	7590 (99.6)	604 (96.8)	8194 (99.4)	**<.001**
Yes	33 (0.4)	20 (3.2)	53 (0.6)	
Disease of the biliary system				
No	7348 (96.4)	588 (94.2)	7936 (96.2)	**.011**
Yes	275 (3.6)	36 (5.8)	311 (3.8)	
Chronic hepatitis				
No	7229 (94.8)	597 (95.7)	7826 (94.9)	.395
Yes	394 (5.2)	27 (4.3)	421 (5.1)	
COPD				
No	7570 (99.3)	618 (99.0)	8188 (99.3)	.453
Yes	53 (0.7)	6 (1.0)	59 (0.7)	

Abbreviations: COPD, chronic obstructive pulmonary disease; DM, diabetes mellitus.

Bold indicates statistically significant values (*P* < .05) .

### 3.2 Nutritional status of the DM vs non‐DM group

**FIGURE 1:**
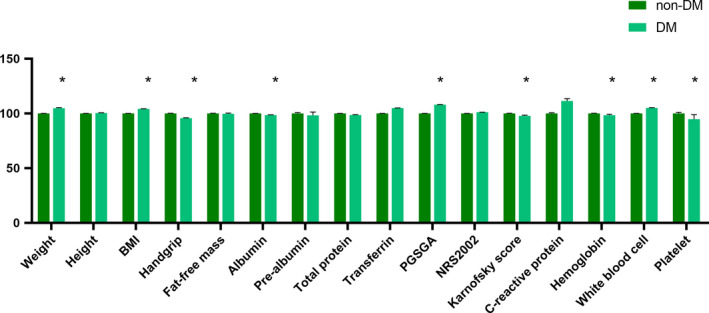
Nutritional status of patients with diabetes compared with those without. All of the parameters were expressed as the means ± SEM with the non‐DM group being set as the control. Inter‐group comparisons were made by *t*‐tests, with *P* < .05 considered to be statistically significant and indicated by an asterisk

**FIGURE 2:**
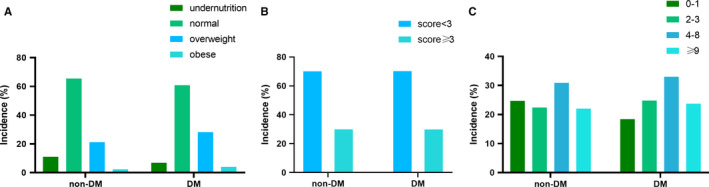
Nutritional patterns of patients with and without diabetes. The nutritional status of each patient was evaluated utilizing three tools, BMI (A), NRS2002 score (B), and PG‐SGA (C). A BMI < 18.5 indicated undernutrition, 18.5‐24.9 normal status, 25‐29.9 overweight and ≥30.0 obesity. For NRS2002 and PG‐SGA, a total score was obtained which categorized the patients into different nutritional status categories. An NRS2002 score ≥3 indicated nutritional risk while a score <3 suggested no risk. The PG‐SGA score classified each participant into one of the following categories: (a) 0‐1, well‐nourished; (b) 2‐3, suspected malnutrition; (c) 4‐8, moderately malnourished; (d) ≥9, severely malnourished. A Chi‐squared test was performed to compare the distribution patterns of the nutritional status between two groups

Next we sought to explore the patterns of nutritional status in patients with diabetes. An inter‐group comparison was conducted concerning pivotal nutritional parameters such as weight, BMI, fat‐free mass, (pre)albumin level, and handgrip. As shown in Figure 1, patients with diabetes had a greater body weight (62.65 kg vs 59.75 kg, *P* < .001). Compared with the non‐DM group, the DM group displayed a similar fat‐free mass (45.09 kg vs 45.22 kg, *P* = .847) yet counterintuitively a lower handgrip (24.50 kg vs 25.61 kg, *P* = .012). In terms of blood indices, the level of albumin (38.38 g/L vs 38.91g/L, *P* = .011) was decreased in the DM group compared to the non‐DM group. The DM group also exhibited a lower level of hemoglobin (121.31 g/L vs 123.29 g/L, *P* = .024) and a lower platelet count (221.54*10^9^/L vs 233.52*10^9^/L, *P* = .005). Notably, an increased PG‐SGA (5.79 vs 5.36, *P* = .030) and a decreased Karnofsky performance score (85.18 vs 87.03, *P* = .002) were observed in the DM group. Compared with the non‐DM group, a higher proportion of patients with diabetes were overweight/obese (Figure 2A, 32.2% vs 23.5%) as indicated by BMI and were more likely to be at risk of malnutrition as evaluated by the PG‐SGA (Figure 2C, score ≥ 4, 56.7% vs 52.9%). There was no statistical significance between two groups when evaluated by NRS2002 (Figure 2B, score ≥ 3, 69.7% vs 69.8%).

### 3.3 Stratified analysis by cancer type

We further characterized the nutritional status of patients with diabetes when they were stratified by the type of malignancy (Table 2). We focused on cancers with high morbidity, which included colorectal, lung, gastric, and breast carcinoma. In the breast cancer subgroup, although the patients with diabetes had a greater body mass (BMI 25.19 vs 24.10, *P* = .004), they showed a compromised handgrip strength (18.34 kg vs 20.38 kg, *P* = .008) and lower Karnofsky performance score. Diabetes patients also had a higher body weight and BMI in the colorectal and lung cancer subgroups. In the lung cancer patients, an elevated CRP level (25.96 mg/L vs 16.84 mg/L, *P* = .016) was observed for patients with DM.

**TABLE 2:** Nutritional status of cancer patients with and without diabetes mellitus

Parameters	Colorectal cancer (N = 1630)			Lung cancer (N = 1571)			Gastric cancer (N = 979)			Breast cancer (N = 1244)		
	Non‐DM	DM	*P*‐value	Non‐DM	DM	*P*‐value	Non‐DM	DM	*P*‐value	Non‐DM	DM	*P*‐value
NRS2002	2.26	1.99	0.062	1.27	1.20	0.626	2.66	2.92	0.244	0.76	0.96	0.090
Karnofsky score	84.99	83.11	0.132	87.01	86.10	0.393	83.31	82.66	0.713	91.49	88.33	**0.004**
Total protein (g/L)	66.74	66.78	0.958	67.99	67.94	0.929	64.67	64.99	0.740	69.69	70.05	0.616
Albumin (g/L)	38.20	37.59	0.162	38.78	38.50	0.559	36.66	35.66	0.123	41.66	41.56	0.847
Pre‐albumin (mg/L)	208.87	209.04	0.979	219.69	216.60	0.641	192.81	192.49	0.971	233.50	241.42	0.214
Transferrin (g/L)	2.30	2.52	0.178	2.27	2.23	0.671	2.38	3.37	**0.003**	2.49	2.34	0.194
C‐reactive protein (mg/L)	20.47	14.36	0.201	16.84	25.96	**0.016**	20.93	31.77	0.221	6.94	9.84	0.469
Hemoglobin (g/L)	120.81	119.28	0.407	126.38	124.29	0.281	116.58	114.97	0.579	123.44	124.52	0.565
White blood cells (*10^9^/L)	6.93	6.98	0.860	6.99	7.30	0.323	6.92	7.11	0.650	6.15	6.35	0.455
Platelets (*10^9^/L)	235.95	216.38	**0.014**	238.27	213.45	**0.006**	239.62	257.97	0.163	242.00	219.20	**0.027**
Fat‐free mass (kg)	46.52	46.96	0.806	47.86	48.00	0.917	46.76	44.45	0.484	41.32	41.21	0.885
Height (cm)	162.30	163.38	0.126	164.71	166.01	0.067	164.10	165.65	0.131	158.25	157.21	0.077
Weight (kg)	59.51	62.92	**<0.001**	61.96	65.56	**<0.001**	57.10	59.73	0.052	60.40	62.33	0.063
BMI	22.52	23.53	**<0.001**	22.76	23.77	**<0.001**	21.15	21.74	0.165	24.10	25.19	**0.004**
Handgrip strength (kg)	26.61	25.49	0.264	26.78	26.41	0.695	26.38	26.38	1.000	20.38	18.34	**0.008**

Abbreviations: BMI, body mass index; DM, diabetes mellitus.

Bold indicates statistically significant values (*P* < .05) .

### 3.4 Survival analyses

**FIGURE 3:**
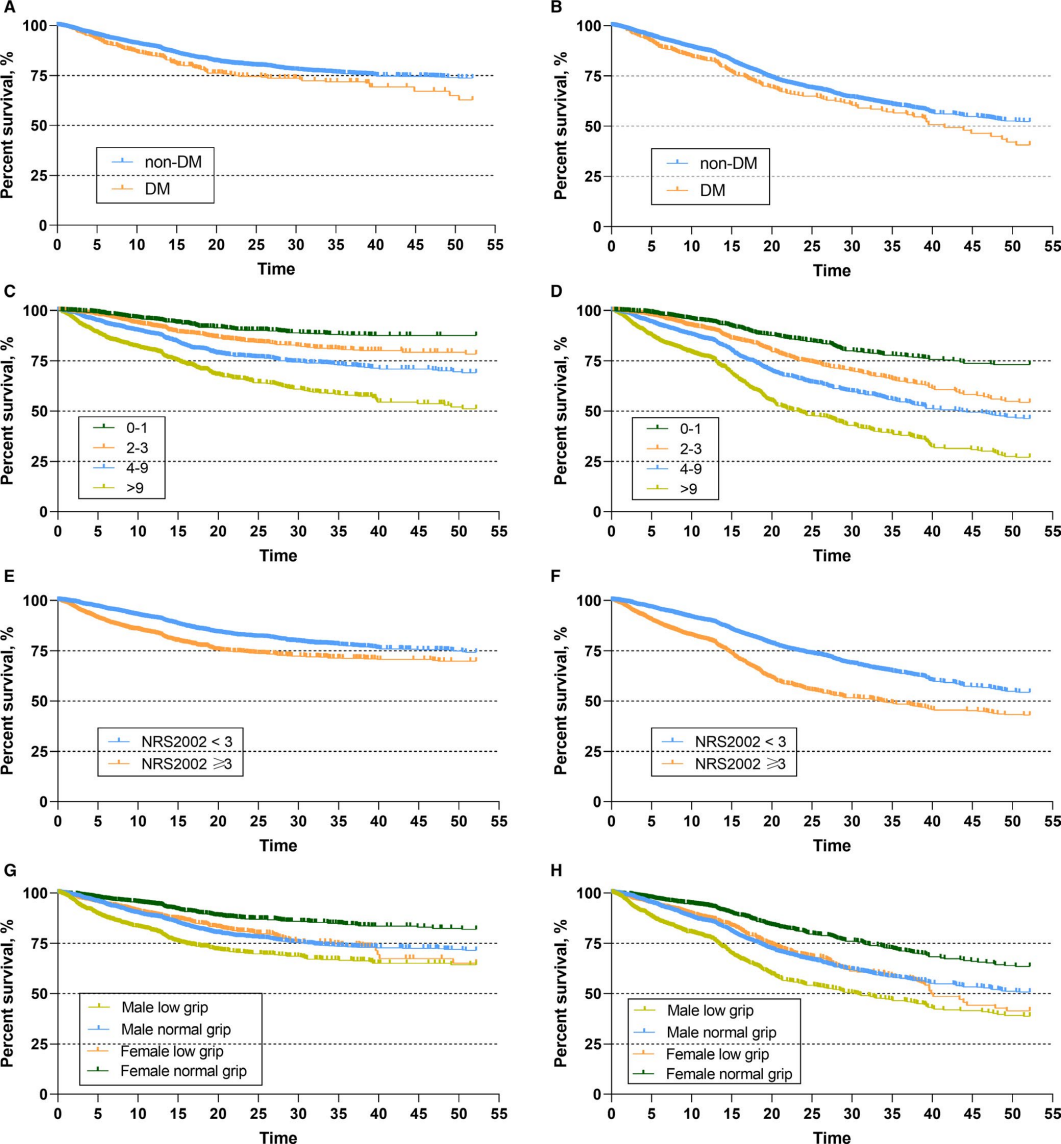
Kaplan‐Meier survival curves of the study population. The CSS (A) and OS (B) for patients with and without DM. The CSS (C) and OS (D) for patients in different nutritional status groups as assessed by PG‐SGA. The CSS (E) and OS (F) for patients in different nutritional status groups as assessed by NRS2002. The CSS (G) and OS (H) for patients with different levels of handgrip strength. Low muscle strength was defined as <18 kg for women and <26 kg for men according to the Asian Working Group for Sarcopenia (AWGS) criterion

The average duration of follow‐up was 24.3 months. As of August 2019, 1944 and 196 deaths had occurred in the non‐DM (N = 7606) and DM group (N = 623), respectively. Additionally, 1244 and 133 cancer‐specific deaths were recorded in the non‐DM and DM groups. Patients with DM showed worse CSS (Figure 3A and 4‐year CSS 62% vs 73%, *P* = .002) and OS (Figure 3B, 4y‐OS 39% vs 52%, *P* = .003) compared with non‐DM patients.

We also evaluated the impact of nutrition‐related factors on the survival of cancer patients. Patients at risk of malnutrition as determined by PG‐SGA had worse outcomes (Figure 3C, 4‐year CSS 51% vs 68% vs 78% vs 88%, *P* < .001; Figure 3D, 4‐year OS 26% vs 45% vs 53% vs 73%, *P* < .001) or NRS2002 score (Figure 3E, 4‐year CSS 70% vs 74%, *P* < .001; Figure 3F, 4‐year OS 43% vs 54%, *P* < .001). Patients with a lower handgrip strength also showed reduced survival compared with patients with a normal handgrip strength, regardless of sex (Figure 3G, 4‐year CSS male 64% vs 71%, female 65% vs 81%, *P* < .001; Figure 3H, 4‐year CSS male 38% vs 50%, female 40% vs 63%, *P* < .001).

Table 3 shows the prognostic value of candidate factors based on a multivariate Cox regression analysis. After correcting for other confounders including sex, the clinical stage, smoking/drinking status, BMI, handgrip strength and treatment received, complication with diabetes was found to be associated with a slightly increased risk of both cancer‐specific (hazard ratio (HR) = 1.282, 95% confidence interval (CI) 1.070‐1.536, *P* = .007) and overall mortality (HR = 1.206, 95% CI 1.040‐1.399, *P* = .013). Regular smoking was predictive of a worse CSS (HR = 1.470, 95% CI 1.279‐1.689, *P* < .001) and OS (HR = 1.318, 95% CI 1.279‐1.689, *P* < .001). Notably, a low handgrip strength was independently predictive of a worse outcome in terms of both CSS (HR = 1.459, 95% CI 1.308‐1.627, *P* < .001) and OS (HR = 1.466, 95% CI 1.342‐1.600, *P* < .001).

**TABLE 3:** Multivariate analysis by Cox proportional hazards regression model

Variables		CSS		OS	
		HR (95% CI)	*P* value	HR (95% CI)	*P* value
Sex	Female	Ref	Ref	Ref	Ref
	Male	1.205 (1.043‐1.393)	.011	1.249 (1.116‐1.398)	<.001
Clinical stage	I‐II	Ref	Ref	Ref	Ref
	III‐IV	1.678 (1.482‐1.899)	<.001	1.826 (1.649‐2.021	<.001
Smoking status	Occasional/never	Ref	Ref	Ref	Ref
	Regular	1.470 (1.279‐1.689)	<.001	1.318 (1.182‐1.469)	<.001
Drinking status	Occasional/never	Ref	Ref	Ref	Ref
	Regular	1.176 (1.027‐1.347)	.019	1.109 (0.992‐1.239)	.069
Area of residence	Rural	Ref	Ref	Ref	Ref
	Urban	1.455 (1.294‐1.636)	<.001	1.335 (1.217‐1.465)	<.001
Education	(Under)graduate	Ref	Ref	Ref	Ref
	Primary/middle/home schooling	1.265 (1.079‐1.483)	.004	1.206 (1.063‐1.368)	.004
Diabetes mellitus	No	Ref	Ref	Ref	Ref
	Yes	1.282 (1.070‐1.536)	.007	1.206 (1.040‐1.399)	.013
BMI	Overweight/obese	Ref	Ref	Ref	Ref
	Normal	1.183 (1.030‐1.359)	.017	1.220 (1.092‐1.362)	<.001
	Underweight	1.884 (1.571‐2.259)	<.001	1.846 (1.591‐2.142)	<.001
Handgrip strength	Normal	Ref	Ref	Ref	Ref
	Low	1.459 (1.308‐1.627)	<.001	1.466 (1.342‐1.600)	<.001
Nutritional treatment	Yes	Ref	Ref	Ref	Ref
	No	1.442 (1.269‐1.639)	<.001	1.361 (1.224‐1.513)	<.001

Given the heterogeneity in the prognosis of different types of malignancies, we further validated the findings derived from the whole study population in each cancer subset. The results of a survival analysis data for six common cancers are shown in Table S1. Complication with diabetes mellitus was associated with a reduced survival only in the breast cancer (Figure S4, 4‐year OS 75% vs 87%, *P* = .025) and esophageal cancer (Figure S4, 4‐year CSS 42% vs 67%, *P* = .026) patients. Among the parameters used to evaluate the nutritional status, a higher PG‐SGA score and lower handgrip strength, which reflect malnourishment or sarcopenia, were consistently associated with worse clinical outcomes in all subsets. However, nutritional risk detected by NRS2002 seemed to have little bearing on the survival of patients with cancers of the digestive tract.

In multivariate analyses which incorporated potential confounders including the TNM stage (Tables S2‐S4), diabetes was again proven to be prognostic in breast cancer (Table S4, for OS, HR = 1.891, 95% CI 1.047‐3.418, *P* = .035) and esophageal cancer (for CSS, HR = 2.903, 95% CI 1.429‐5.897, *P* = .003; for OS, HR = 2.100, 95% CI 1.048‐4.206, *P* = .036) patients. Being underweight (as assessed by BMI) and having a lower handgrip strength were both independent prognostic factors in colorectal, lung and nasopharyngeal cancer patients. Nutritional treatment seemed to have a positive effect (boundary effect estimates ranging from 1.2 to 1.85) on the survival of patients with cancers of the lung and digestive tract (esophagus, stomach, and colorectum).

## 4 DISCUSSION

In this large‐scale prospective study, we found that cancer patients with diabetes had a greater body mass yet counterintuitively had a lower handgrip strength, reduced level of serum albumin and poorer performance status when compared with non‐DM patients. Although a higher proportion of patients with DM were overweight/obese, DM was also related to a higher risk of malnutrition. Complication with diabetes was predictive of increased cancer‐specific and overall mortality.

The clinical relevance of malnutrition in cancer patients has long been recognized. Previous studies identified undernutrition as a negative prognostic factor in patients with malignancies.^12^, ^13^ As a metabolic abnormality‐related disease, diabetes is also a common contributor to nutritional disorders. The number of cancer cases complicated with diabetes has kept climbing due to the growing populations of cancer survivors and diabetes patients. Notwithstanding the conceivable diversity of nutritional status among these patients, few studies have been done to characterize the patterns.

Our present study revealed that cancer patients with diabetes had a higher body weight and were more likely to have an excessive body mass, which agreed with previous studies and supported the assertion that overweight/obesity is an important risk factor for developing diabetes.^9^ It is noteworthy that even among those with an increased body mass and a similar fat‐free mass, patients with diabetes demonstrated decreased muscle strength. Patients with diabetes had a higher proportion of adipose tissue and depleted muscle mass, which thus led to a decreased functional capacity, so these patients were fatter and weaker than the non‐DM patients. This clinical condition is associated with sarcopenic obesity, which is indicative of a poorer performance status and the reduced survival of cancer patients.^10^

The mechanism underlying the connection between diabetes and sarcopenic obesity might be extensive metabolic aberrations (insulin resistance and disrupted glucose utilization). More importantly, obesity and diabetes are co‐contributors to a persistent low‐grade inflammatory response.^14^ Presumably, interactions are present between glucolipotoxicity and stress‐related hormones, the latter leading to a multi‐faceted syndrome characterized by inflammation and abnormal metabolic function.^15^, ^16^ Diabetes mellitus also features dysregulated innate immunity, as manifested by the activation of cytokines, chemokines, and co‐stimulatory molecules in peripheral blood monocytes.^17^, ^18^ These inflammatory mediators augment protein catabolism and the loss of skeletal muscle mass.^11^ This can be evidenced by the decreased albumin and elevated CRP level observed in the current study.

Consistent with previous studies, our study found that DM was associated with compromised survival. Presumably, DM is related to both an elevated risk of cancer as well as cancer‐specific death.^11^, ^15^, ^16^, ^17^, ^18^ Hyperinsulinemia and subsequent pro‐cancerous cellular signaling are both considered to underlie the poorer prognosis in DM patients.^14^ The cancer patients with DM tended to be overweight/obese yet weaker in muscle strength, both of which were proven to be prognostic factors in our study. To assess the influence of DM on the overall survival, we performed a multivariate regression analysis to adjust for the influence of other potential covariates, and found that DM was independently predictive of a worse OS. Although the prognostic effect of diabetes was observed only in the breast and esophageal carcinoma patients in a subsequent analysis, the lack of statistical significance for other types may be due to the insufficient sample size of the DM group (ranging from 22 to 162 individuals) in each cancer subset.

Moreover we found that the diabetes group tended to have more medical complications such as hypertension, cardiovascular disease and stroke, to name a few, all of which could contribute to and aggravate physical decline. Another finding was the high prevalence of diabetes in pancreatic cancer patients. Diabetes has been indicated as a risk factor for malignancy, and our data provided hints about the link between diabetes and cancer, especially pancreatic cancer.

Although there have been previous studies that have suggested that DM was associated with the prognosis of several cancers, the present study has several improvements in comparison with earlier studies. First and foremost, this was a prospective study with a large number of subjects generating nationally‐representative data. Second, we provided initial evidence that cancer patients with diabetes had a unique nutritional condition, which could not be simply categorized as malnutrition by excess or deficit. Another noteworthy finding was that a lower handgrip strength independently indicated worse survival in multiple common cancers, and nutritional treatment of any form might have protective effects in patients with cancers of the digestive tract. This was clinically important considering the universal under‐awareness of nutritional evaluation and intervention among medical staff. An abnormally low handgrip strength probably reflects malnourishment or even preclinical cachexia, and nutritional treatment should be prescribed when necessary.

Nevertheless, the present study does have limitations. As with all observational research, unrandomized grouping might have led to some selection bias, which we tried to control via multi‐center recruitment of participants. Also, unmeasured covariates might have exerted confounding effects. In addition, we did not measure the body composition, and thus failed to precisely identify the patients with sarcopenia. Further study incorporating muscle mass assessment methods, such as CT scanning, is warranted.

## 5 CONCLUSIONS

Compared with those without diabetes, cancer patients with diabetes had a larger body mass yet lower muscle strength, poorer performance status and higher incidence of malnourishment, which was accompanied by a decreased level of albumin. Complication with diabetes was associated with compromised survival. Tailored nutritional intervention is necessary for this subpopulation considering its unique pattern of nutritional status.

## CONFLICT OF INTEREST

The authors disclose no potential conflicts of interest.

## AUTHOR CONTRIBUTIONS

Minghua Cong contributed to data curation, formal analysis, writing–original draft, and writing–review and editing. Wenjie Zhu contributed to data curation, formal analysis, methodology, software, writing–original draft, and writing–review and editing. Chang Wang, Zhenming Fu, Zhong Dai, Keqing Yao, Zengqing Guo, Yuan Lin, Yingying Shi, Wen Hu, Yi Ba, Suyi Li, Zengning Li, Kunhua Wang, Jing Wu, Ying He, Jiajun Yang, Conghua Xie, Xinxia Song, Gongyan Chen, Wenjun Ma, Suxia Luo, Zihua Chen, Hu Ma, Chunling Zhou, Wei Wang, Qi Luo, Yongmei Shi, Yumei Qi, Haiping Jiang, Wenxian Guan, Junqiang Chen, Jiaxin Chen, Yu Fang, Lan Zhou, Yongdong Feng, Rongshao Tan, Tao Li, Junwen Ou, Qingchuan Zhao, Jianxiong Wu, Li Deng, Xin Lin, and Liuqing Yang contributed to investigation, methodology, and writing–review and editing.. Chunhua Song contributed to investigation, formal analysis, methodology, and writing–review and editing....... Hongxia Xu contributed to conceptualization, data curation, and writing–review and editing. Wei Li contributed to investigation, methodology, data curation, and writing–review and editing. Lei Yu contributed to investigation, methodology, data curation, supervision, and writing–review and editing. Hanping Shi contributed to conceptualization, funding acquisition, supervision, data curation, and writing–review and editing.

## Supporting information

Fig S1

Fig S2

Fig S3

Fig S4

Supplementary Material

## Data Availability

The data described in the manuscript will be made available upon request.
