# Supplementary figures and images for: Nutritional status and survival of 8247 cancer patients with or without diabetes mellitus—results from a prospective cohort study
Source: Cancer Med. 2020 Aug 19;9(20):7428–39. doi: 10.1002/cam4.3397 (PMC7571830; doi:10.1002/cam4.3397)

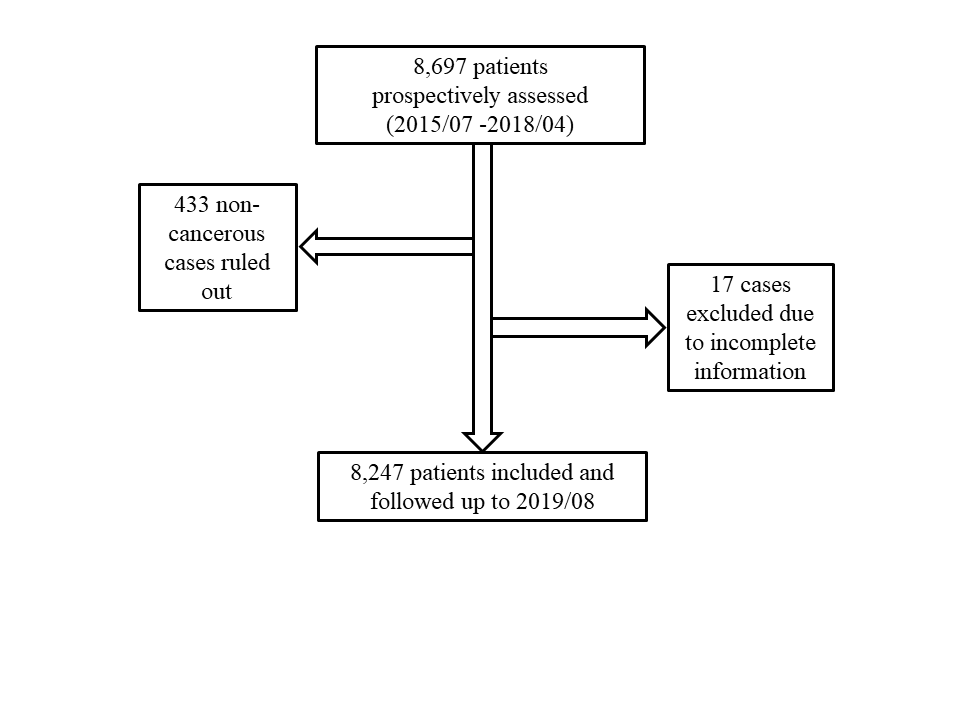

Supplement: Supplementary file 1 — Fig S1 [file CAM4-9-7428-s001.tif]

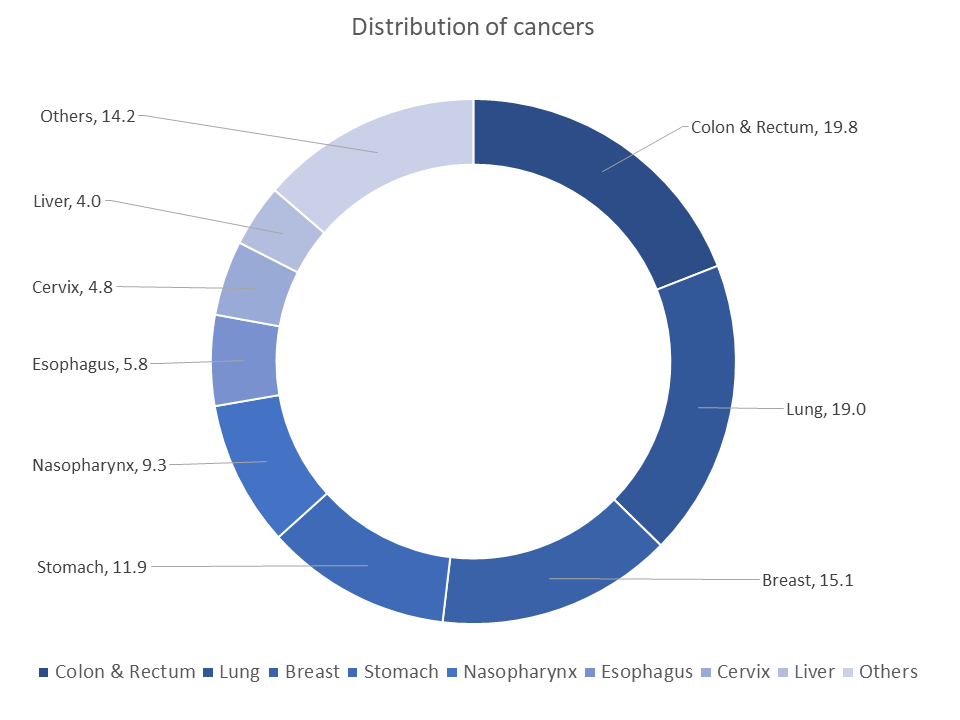

Supplement: Supplementary file 2 — Fig S2 [file CAM4-9-7428-s002.TIF]

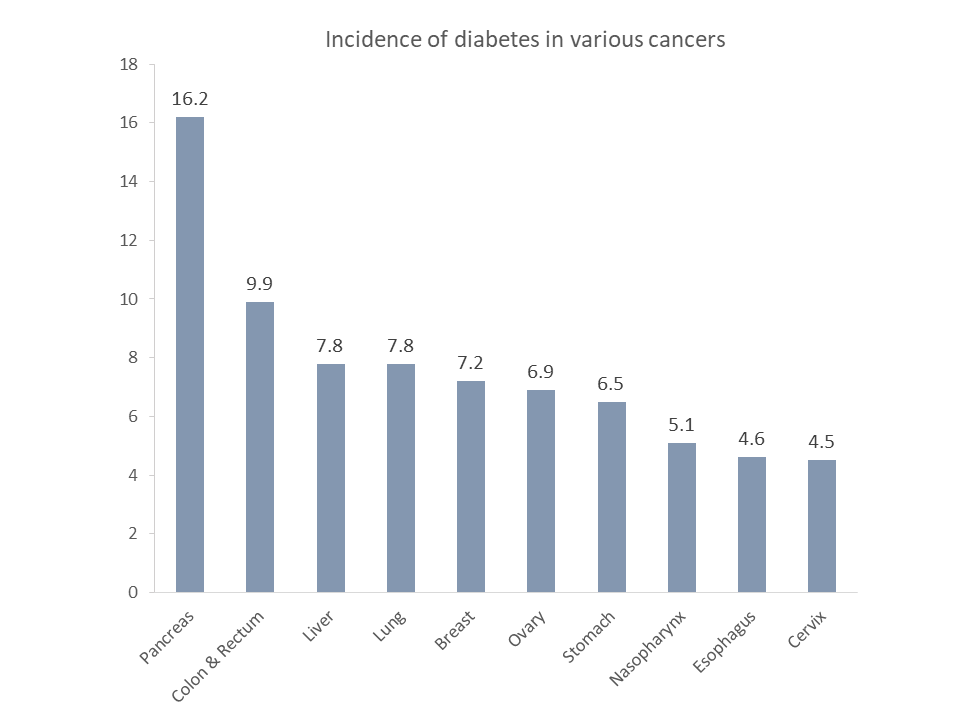

Supplement: Supplementary file 3 — Fig S3 [file CAM4-9-7428-s003.TIF]

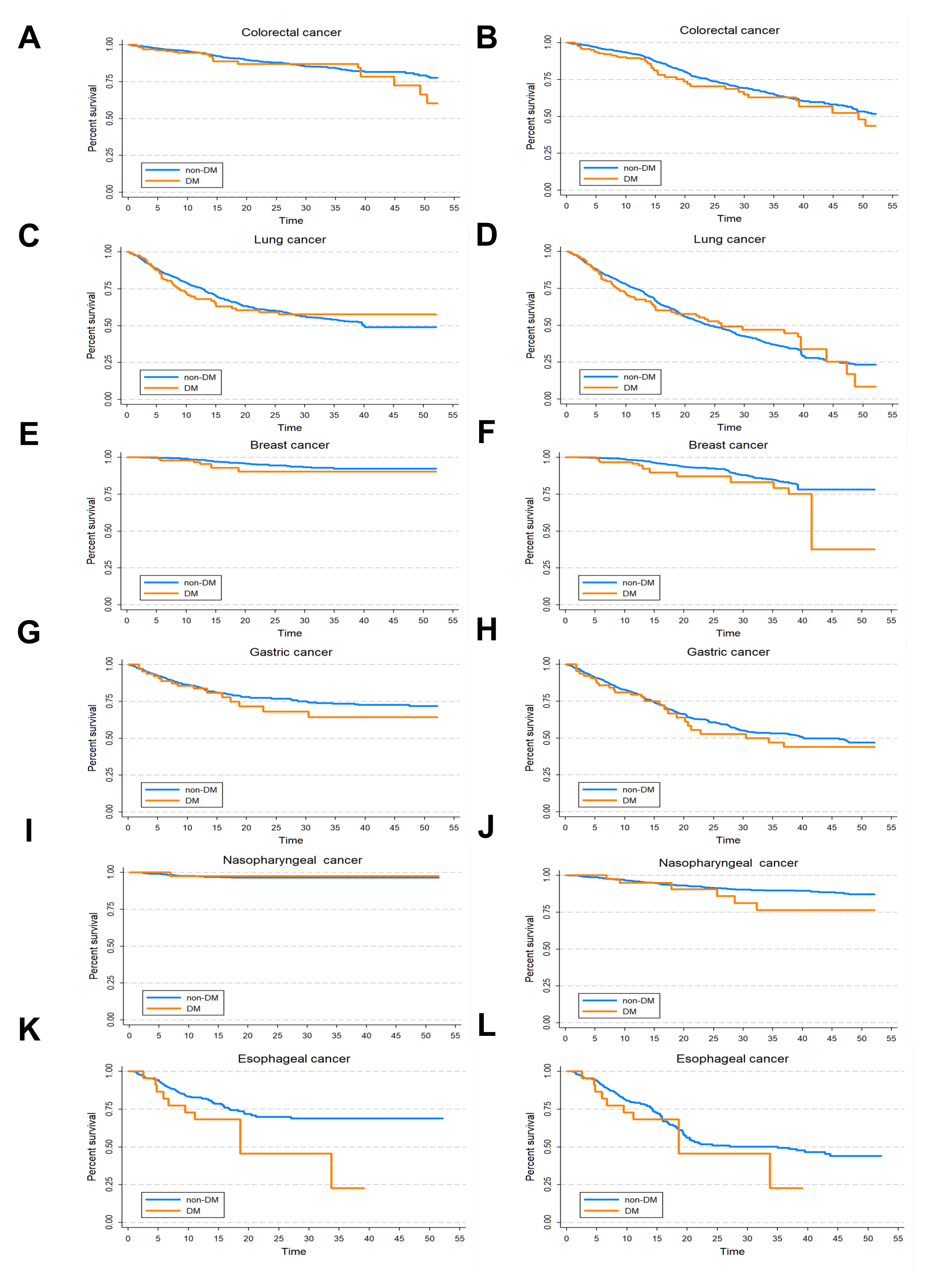

Supplement: Supplementary file 4 — Fig S4 [file CAM4-9-7428-s004.tif]
