# Supplementary material for: Nutritional status and survival of 8247 cancer patients with or without diabetes mellitus—results from a prospective cohort study
Source: Cancer Med. 2020 Aug 19;9(20):7428–39. doi: 10.1002/cam4.3397 (PMC7571830; doi:10.1002/cam4.3397)
Supplement: Supplementary file 5 — Supplementary Material [file CAM4-9-7428-s005.docx]

Table S1. Survival analysis in patients with six common malignancies

| **Type of cancer** | **Parameter** | **Group** | **Endpoints** | | | |
| --- | --- | --- | --- | --- | --- | --- |
|  |  |  | 4-year CSS (%) | Log-rank p value | 4-year OS (%) | Log-rank p value |
| Colorectal cancer (n=1,618) | Diabetes mellitus | non-DM  (n=1,456) | 83.6 | 0.440 | 68.2 | 0.346 |
|  |  | DM (n=162) | 82.1 |  | 64.8 |  |
|  | PG-SGA | 0-1 (n=226) | 87.8 | **<0.001** | 65.8 | **<0.001** |
|  |  | 2-3 (n=339) | 84.0 |  | 61.0 |  |
|  |  | 4-9 (n=609) | 79.5 |  | 51.7 |  |
|  |  | >9 (n=444) | 52.5 |  | 31.7 |  |
|  | NRS2002 | <3 (n=956) | 70.7 | 0.929 | 49.9 | **0.012** |
|  |  | ≥3 (n=662) | 80.9 |  | 49.3 |  |
|  | Handgrip | Male low (n=346) | 70.6 | **0.023** | 39.4 | **<0.001** |
|  |  | Male normal (n=604) | 79.8 |  | 58.5 |  |
|  |  | Female low (n=291) | 62.6 | **<0.001** | 39.9 | **<0.001** |
|  |  | Female normal (n=377) | 80.1 |  | 55.7 |  |
| Lung cancer (n=1,570) | Diabetes mellitus | non-DM  (n=1,447) | 48.2 | 0.656 | 35.8 | 0.974 |
|  |  | DM (n=123) | 45.4 |  | 35.1 |  |
|  | PG-SGA | 0-1 (n=359) | 61.1 | **<0.001** | 28.4 | **<0.001** |
|  |  | 2-3 (n=433) | 63.2 |  | 29.7 |  |
|  |  | 4-9 (n=483) | 41.6 |  | 23.1 |  |
|  |  | >9 (n=295) | 24.1 |  | 5.4 |  |
|  | NRS2002 | <3 (n=1,281) | 51.8 | **<0.001** | 36.9 | **<0.001** |
|  |  | ≥3 (n=289) | 41.8 |  | 23.8 |  |
|  | Handgrip | Male low (n=377) | 49.2 | **0.006** | 34.0 | **0.043** |
|  |  | Male normal (n=691) | 51.7 |  | 35.2 |  |
|  |  | Female low (n=194) | 38.5 | **0.001** | 16.4 | **0.002** |
|  |  | Female normal (n=308) | 63.0 |  | 34.6 |  |
| Breast cancer (n=1,244) | Diabetes mellitus | non-DM  (n=1,153) | 92.8 | 0.197 | 87.1 | **0.025** |
|  |  | DM (n=91) | 87.5 |  | 74.8 |  |
|  | PG-SGA | 0-1 (n=443) | 93.9 | **<0.001** | 80.2 | **<0.001** |
|  |  | 2-3 (n=395) | 95.5 |  | 90.4 |  |
|  |  | 4-9 (n=313) | 91.1 |  | 77.4 |  |
|  |  | >9 (n=93) | 75.0 |  | 66.8 |  |
|  | NRS2002 | <3 (n=1,142) | 93.0 | **0.006** | 75.1 | **0.005** |
|  |  | ≥3 (n=102) | 78.0 |  | 59.1 |  |
|  | Handgrip | Male low (n=3) | NA | NA | NA | NA |
|  |  | Male normal (n=2) | NA |  | NA |  |
|  |  | Female low (n=463) | 90.7 | 0.157 | 77.1 | **0.028** |
|  |  | Female normal (n=776) | 93.0 |  | 82.4 |  |
| Gastric cancer (n=975) | Diabetes mellitus | non-DM  (n=911) | 67.8 | 0.504 | 51.2 | 0.576 |
|  |  | DM (n=64) | 62.8 |  | 45.8 |  |
|  | PG-SGA | 0-1 (n=59) | 87.3 | **0.007** | 72.0 | **<0.001** |
|  |  | 2-3 (n=120) | 81.7 |  | 54.2 |  |
|  |  | 4-9 (n=392) | 72.5 |  | 52.0 |  |
|  |  | >9 (n=404) | 64.4 |  | 33.9 |  |
|  | NRS2002 | <3 (n=411) | 66.6 | 0.822 | 42.8 | 0.436 |
|  |  | ≥3 (n=564) | 74.8 |  | 48.4 |  |
|  | Handgrip | Male low (n=272) | 68.0 | **0.013** | 31.6 | **<0.001** |
|  |  | Male normal (n=418) | 72.4 |  | 52.4 |  |
|  |  | Female low (n=135) | 71.8 | 0.869 | 45.2 | 0.912 |
|  |  | Female normal (n=150) | 74.2 |  | 52.6 |  |
| Nasopharyngeal cancer (n=766) | Diabetes mellitus | non-DM  (n=727) | 94.7 | 0.774 | 87.0 | 0.142 |
|  |  | DM (n=39) | 95.7 |  | 76.9 |  |
|  | PG-SGA | 0-1 (n=494) | 98.3 | **<0.001** | 91.1 | **<0.001** |
|  |  | 2-3 (n=132) | 94.7 |  | 74.2 |  |
|  |  | 4-9 (n=98) | 94.8 |  | 80.2 |  |
|  |  | >9 (n=42) | 65.8 |  | 40.5 |  |
|  | NRS2002 | <3 (n=664) | 97.2 | **0.005** | 88.2 | **<0.001** |
|  |  | ≥3 (n=102) | 90.6 |  | 73.0 |  |
|  | Handgrip | Male low (n=103) | 89.6 | **0.001** | 72.2 | **0.002** |
|  |  | Male normal (n=467) | 97.3 |  | 87.9 |  |
|  |  | Female low (n=37) | 88.7 | **<0.001** | 67.8 | **<0.001** |
|  |  | Female normal (n=159) | 100.0 |  | 95.5 |  |
| Esophageal cancer (n=480) | Diabetes mellitus | non-DM  (n=458) | 66.6 | **0.026** | 54.4 | 0.187 |
|  |  | DM (n=22) | 41.9 |  | 41.9 |  |
|  | PG-SGA | 0-1 (n=30) | 80.6 | **0.040** | 48.3 | **0.031** |
|  |  | 2-3 (n=48) | 71.8 |  | 61.1 |  |
|  |  | 4-9 (n=216) | 69.7 |  | 46.5 |  |
|  |  | >9 (n=186) | 59.7 |  | 34.9 |  |
|  | NRS2002 | <3 (n=205) | 71.6 | 0.073 | 45.9 | 0.106 |
|  |  | ≥3 (n=275) | 63.1 |  | 40.8 |  |
|  | Handgrip | Male low (n=159) | 63.3 | **0.001** | 40.0 | 0.379 |
|  |  | Male normal (n=241) | 67.9 |  | 43.7 |  |
|  |  | Female low (n=32) | 70.6 | **<0.001** | 25.0 | **<0.001** |
|  |  | Female normal (n=48) | 76.4 |  | 66.8 |  |

Table S2. Multivariate analysis in patients with colorectal cancer

| **Variables** | | **CSS** | | **OS** | |
| --- | --- | --- | --- | --- | --- |
|  |  | **HR (95% CI)** | **p value** | **HR (95% CI)** | **p value** |
| Sex | Female | Ref | Ref | Ref | Ref |
|  | Male | 0.868 (0.577-1.305) | 0.495 | 0.967 (0.736-1.269) | 0.806 |
| Clinical stage | I-II | Ref | Ref | Ref | Ref |
|  | III-IV | 1.494 (1.029-2.169) | **0.035** | 1.647 (1.269-2.138) | **<0.001** |
| Smoking status | Occasional/Never | Ref | Ref | Ref | Ref |
|  | Regular | 1.174 (0.784-1.758) | 0.436 | 1.285 (0.979-1.685) | 0.070 |
| Drinking status | Occasional/Never | Ref | Ref | Ref | Ref |
|  | Regular | 1.354 (0.888-2.064) | 0.159 | 0.872 (0.643-1.182) | 0.377 |
| Area of residence | Rural | Ref | Ref | Ref | Ref |
|  | Urban | 1.418 (1.004-2.000) | **0.047** | 1.165 (0.925-1.466) | 0.194 |
| Education | (Under)graduate | Ref | Ref | Ref | Ref |
|  | Primary/middle/ home schooling | 1.232 (0.784-1.937) | 0.365 | 1.302 (0.946-1.792) | 0.162 |
| Diabetes mellitus | No | Ref | Ref | Ref | Ref |
|  | Yes | 1.242 (0.762-2.025) | 0.384 | 1.225 (0.872-1.720) | 0.242 |
| BMI | Overweight/obese | Ref | Ref | Ref | Ref |
|  | Normal | 0.968 (0.656-1.428) | **0.027** | 1.033 (0.792-1.346) | 0.813 |
|  | Underweight | 1.556 (0.928 -2.608) | 0.094 | 1.490 (1.027-2.162) | **0.036** |
| Handgrip | Normal | Ref | Ref | Ref | Ref |
|  | Low | 1.762 (1.279-2.427) | **0.001** | 1.643 (1.320-2.047) | **<0.001** |
| Nutritional treatment | Yes | Ref | Ref | Ref | Ref |
|  | No | 1.851 (1.328-2.580) | **<0.001** | 1.368 (1.073-1.746) | **0.012** |

Table S3. Multivariate analysis in patients with lung cancer

| **Variables** | | **CSS** | | **OS** | |
| --- | --- | --- | --- | --- | --- |
|  |  | **HR (95% CI)** | **p value** | **HR (95% CI)** | **p value** |
| Sex | Female | Ref | Ref | Ref | Ref |
|  | Male | 1.232 (0.989-1.536) | 0.063 | 1.255 (1.040-1.515) | **0.018** |
| Clinical stage | I-II | Ref | Ref | Ref | Ref |
|  | III-IV | 1.857 (1.504-2.293) | **<0.001** | 1.927 (1.611-2.306) | **<0.001** |
| Smoking status | Occasional/Never | Ref | Ref | Ref | Ref |
|  | Regular | 1.061 (0.859-1.310) | 0.582 | 1.006 (0.841-1.204) | 0.946 |
| Drinking status | Occasional/Never | Ref | Ref | Ref | Ref |
|  | Regular | 0.994 (0.812-1.217) | 0. 953 | 1.056 (0.887-1.258) | 0.541 |
| Area of residence | Rural | Ref | Ref | Ref | Ref |
|  | Urban | 1.160 (0.962-1.399) | 0.199 | 1.103 (0.939-1.294) | 0.232 |
| Education | (Under)graduate | Ref | Ref | Ref | Ref |
|  | Primary/middle/ home schooling | 1.418 (1.105-1.819) | **0.006** | 1.230 (1.001-1.511) | **0.049** |
| Diabetes mellitus | No | Ref | Ref | Ref | Ref |
|  | Yes | 1.092 (0.806-1.478) | 0.571 | 1.031 (0.790-1.344) | 0.824 |
| BMI | Overweight/obese | Ref | Ref | Ref | Ref |
|  | Normal | 1.165 (0.948-1.432) | 0.145 | 1.218 (1.021-1.454) | **0.029** |
|  | Underweight | 1.705 (1.237 -2.350) | **0.001** | 1.793 (1.350-2.382) | **<0.001** |
| Handgrip | Normal | Ref | Ref | Ref | Ref |
|  | Low | 1.353 (1.142-1.604) | **<0.001** | 1.229 (1.060-1.425) | **0.006** |
| Nutritional treatment | Yes | Ref | Ref | Ref | Ref |
|  | No | 1.387 (1.099-1.750) | **0.006** | 1.234 (0.998-1.527) | 0.052 |

Table S4. Multivariate analysis in patients with breast cancer

| **Variables** | | **CSS** | | **OS** | |
| --- | --- | --- | --- | --- | --- |
|  |  | **HR (95% CI)** | **p value** | **HR (95% CI)** | **p value** |
| Clinical stage | I-II | Ref | Ref | Ref | Ref |
|  | III-IV | 3.614 (2.004-6.516) | **<0.001** | 3.817 (2.478-5.878) | **<0.001** |
| Smoking status | Occasional/Never | Ref | Ref | Ref | Ref |
|  | Regular | 1.428 (0.553-3.684) | 0.461 | 0.927 (0.399-2.151) | 0.860 |
| Drinking status | Occasional/Never | Ref | Ref | Ref | Ref |
|  | Regular | 2.112 (0.502-8.878) | 0. 308 | 0.960 (0.233-3.961) | 0.955 |
| Area of residence | Rural | Ref | Ref | Ref | Ref |
|  | Urban | 1.152 (0.599-2.214) | 0.672 | 0.946 (0.589-1.518) | 0.817 |
| Education | (Under)graduate | Ref | Ref | Ref | Ref |
|  | Primary/middle/ home schooling | 2.251 (0.933-5.429) | 0.071 | 2.207 (1.152-4.230) | **0.017** |
| Diabetes mellitus | No | Ref | Ref | Ref | Ref |
|  | Yes | 1.708 (0.707-4.128) | 0.234 | 1.891 (1.047-3.418) | **0.035** |
| BMI | Overweight/obese | Ref | Ref | Ref | Ref |
|  | Normal | 1.800 (0.952-3.402) | 0.071 | 1.257 (0.819-1.930) | 0.295 |
|  | Underweight | 3.809 (1.227-11.828) | **0.021** | 1.426 (0.497-4.096) | 0.510 |
| Handgrip | Normal | Ref | Ref | Ref | Ref |
|  | Low | 1.306 (0.741-2.300) | 0.356 | 1.412 (0.933-2.137) | 0.103 |
| Nutritional treatment | Yes | Ref | Ref | Ref | Ref |
|  | No | 0.988 (0.439-2.226) | 0.977 | 1.019 (0.571-1.820) | 0.948 |
